# Gene Losses and Variations in Chloroplast Genome of Parasitic Plant *Macrosolen* and Phylogenetic Relationships within Santalales

**DOI:** 10.3390/ijms20225812

**Published:** 2019-11-19

**Authors:** Liping Nie, Yingxian Cui, Liwei Wu, Jianguo Zhou, Zhichao Xu, Yonghua Li, Xiwen Li, Yu Wang, Hui Yao

**Affiliations:** 1 Key Lab of Chinese Medicine Resources Conservation, State Administration of Traditional Chinese Medicine of the People’s Republic of China, Institute of Medicinal Plant Development, Chinese Academy of Medical Sciences and Peking Union Medical College, Beijing 100193, China; nielpforever@sina.com (L.N.); yxcui2017@163.com (Y.C.); 15895996332@163.com (L.W.); jgzhou1316@163.com (J.Z.); xuzhichao830@126.com (Z.X.); ywang@implad.ac.cn (Y.W.); 2 Engineering Research Center of Chinese Medicine Resources, Ministry of Education, Beijing 100193, China; 3 College of Pharmacy, Guangxi University of Traditional Chinese Medicine, Nanning 530200, China; 4 Institute of Chinese Materia Medica, China Academy of Chinese Medical Sciences, Beijing 100700, China; xwli@icmm.ac.cn

**Keywords:** *Macrosolen*, *Macrosolen cochinchinensis*, *Macrosolen tricolor*, *Macrosolen bibracteolatus*, Santalales, gene loss, chloroplast genome, phylogenetic relationship

## Abstract

*Macrosolen* plants are parasitic shrubs, several of which are important medicinal plants, that are used as folk medicine in some provinces of China. However, reports on *Macrosolen* are limited. In this study, the complete chloroplast genome sequences of *Macrosolen cochinchinensis*, *Macrosolen tricolor* and *Macrosolen bibracteolatus* are reported. The chloroplast genomes were sequenced by Illumina HiSeq X. The length of the chloroplast genomes ranged from 129,570 bp (*M. cochinchinensis*) to 126,621 bp (*M. tricolor*), with a total of 113 genes, including 35 tRNA, eight rRNA, 68 protein-coding genes, and two pseudogenes (*ycf1* and *rpl2*). The simple sequence repeats are mainly comprised of A/T mononucleotide repeats. Comparative genome analyses of the three species detected the most divergent regions in the non-coding spacers. Phylogenetic analyses using maximum parsimony and maximum likelihood strongly supported the idea that Loranthaceae and Viscaceae are monophyletic clades. The data obtained in this study are beneficial for further investigations of *Macrosolen* in respect to evolution and molecular identification.

## 1. Introduction

The traits of trophic specialization in all parasitic plants are described as “parasitic reduction syndrome”. At the genetic level, parasitic reduction syndrome includes the functional and physical reduction of heterotrophs’ plastid genomes, where rampant gene loss and an acceleration of molecular evolutionary rates occur [1,2]. Considering the partial or complete absence of their photosynthetic capacity, parasitic plants have to absorb organic nutrients, inorganic nutrients, and water from their hosts [3]. Most parasitic plants are included in the order Santalales and the families Orobanchaceae and Orchidaceae [2]. The first complete chloroplast genome of a parasitic plant was obtained from *Epifagus virginiana*, and all of its photosynthesis and energy producing genes have been lost [4]. Petersen et al. reported the complete plastome sequences of one species of *Osyris* and three species of *Viscum*. These researchers found that these four species have experienced rearrangements, and a number of protein-coding genes and two tRNA genes have been pseudogenised or completely lost [5]. The complete chloroplast genome of *Schoepfia jasminodora* has been reported; *S. jasminodora* represents the early stages of chloroplast genome degradation along with its transition to heterotrophy in related taxa [6]. Li et al. determined the complete chloroplast genome sequences of *Taxillus chinensis* and *Taxillus sutchuenensis*. The results showed that all *ndh* genes, three ribosomal protein genes, seven tRNA genes, four *ycf* genes, and the *infA* gene of these two species have been lost [7]. Previous studies have reported that *Rafflesia lagascae* only contains small fragments of plastid sequences at low coverage depth, and they cannot recover any substantial portions of the chloroplast genome [8]. In the parasitic family Orobanchaceae, the complete chloroplast genomes of some species, including *Cistanche deserticola* [9], *Aureolaria virginica*, *Lindenbergia philippensis* [10], and *Lathraea squamaria* [11], have been reported. These chloroplast genomes have shown physical and functional gene loss or pseudogenization. The *Balanophora* plastid genomes of *Balanophora laxiflora* and *Balanophora reflexa* [12], at 15.5 kb in size with only 19 genes, are the most reduced plastomes reported thus far, except for the 11.3 and 15.2 kb genomes of two holoparasitic species of *Pilostyles* [13] and the 12.8 kb genome of the myco-heterotroph *Sciaphila thaidanica* [14]. *Rhopalocnemis phalloides* [15], which belongs to the family Balanophoraceae, has also shown highly plastid genome reduction with 18.6 kb in length. In addition, gene loss has also been found in myco-heterotrophs [16], where carbon is obtained from fungi, thus forming mycorrhizal symbiosis with their roots. Photosynthesis-related genes are lost first, followed by housekeeping genes, which eventually results in a highly reduced genome [17].

The chloroplast is an important organelle in plant cells, and it primarily carries out photosynthesis and carbon fixation. The chloroplast genome is independent of nuclear genes, and the chloroplast possesses its own independent transcription and transport system [18,19]. A typical chloroplast genome of most angiosperms consists of four parts, namely a pair of inverted repeats (IRa and IRb), a large single-copy (LSC) region and a small single-copy (SSC) region [20]. The chloroplast genome sequences are highly conserved in gene order and content [21], and they are thus ideal research models for the study of molecular markers [22,23], species identification [24,25,26], and species evolution [27]. 

**Figure 1:**
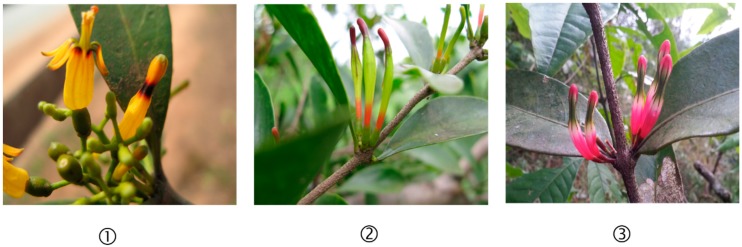
Plant materials of three *Macrosolen* species. ① *Macrosolen cochinchinensis*; ② *Macrosolen tricolor*; and ③ *Macrosolen bibracteolatus*.

*Macrosolen* plants are parasitic shrubs that belong to the family Loranthaceae. There are approximately 40 species of *Macrosolen,* and most of them are distributed in Southern and Southeastern Asia, whereas five species of *Macrosolen* are dispersed in China [28]. *Macrosolen cochinchinensis*, *Macrosolen tricolor,* and *Macrosolen bibracteolatus* have been used as folk medicines in China for a long time. *M. cochinchinensis* is used to clear heat and fire, remove blood stasis, and relieve pain. *M. tricolor* is used to dissipate heat and relieve coughing. *M. bibracteolatus* is used to invigorate the liver and kidney, expel wind, remove dampness, and strengthen tendons and bones [29,30,31]. These species exhibit different medicinal effects. However, they have similar morphologies when they are not in fluorescence (Figure 1), resulting in an extreme difficulty in their identification on the basis of morphological features. The limited reports on *Macrosolen* hinder the related research and development. In this study, we determined the complete chloroplast genome sequences of *M. cochinchinensis*, *M. tricolor* and *M. bibracteolatus*. To reveal the phylogenetic positions of the three species and the evolution of *Macrosolen* within Santalales, we conducted phylogenetic trees using the maximum parsimony (MP) and maximum likelihood (ML) methods on the basis of common protein-coding genes from 16 species. Our results can provide important genetic resources for the study of *Macrosolen*.

## 2. Results

### 2.1. Complete Chloroplast Genomes of Three Macrosolen Species

**Figure 2:**
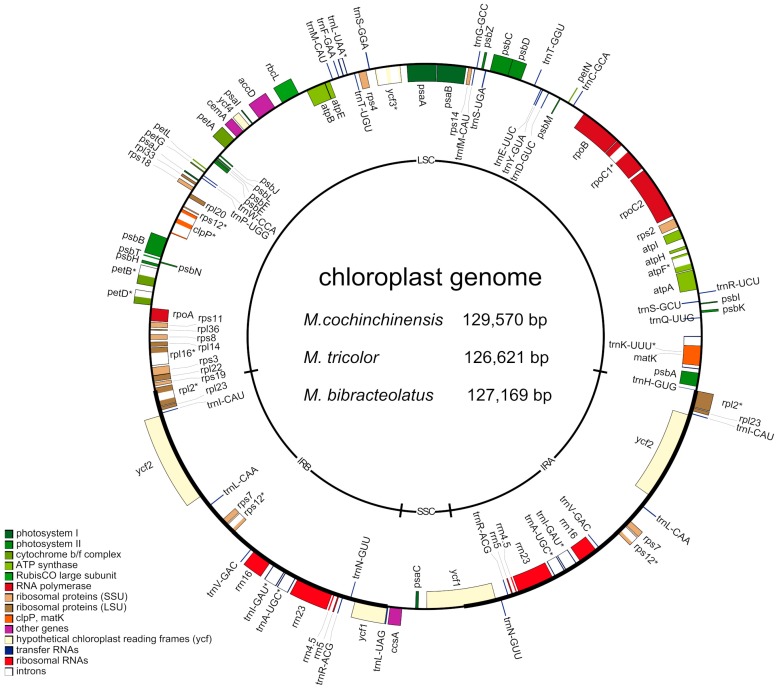
Gene map of the complete chloroplast genome of three *Macrosolen* species. Genes outside the large ring circle are transcribed in a counter-clockwise direction, and genes inside the circle are transcribed clockwise. The same color represents the same category of genes. Deep grey in the inner circle represents GC content, and lighter grey represents A/T content.

The length of the three studied chloroplast genomes ranged from 129,570 bp (*M. cochinchinensis*) to 126,621 bp (*M. tricolor*) with a typical quadripartite structure consisting of a pair of IRs (24,703–25,445 bp) separated by the LSC (70,692–73,052 bp) and the SSC (5320–5724 bp) regions (Figure 2). The three chloroplast genomes were found to highly conserved in GC content, gene content and gene order (Table 1 and Table S1). All three species comprised 113 genes, including 68 protein-coding genes, 35 tRNAs, eight rRNAs and two pseudogenes (*rps12* and *ycf2*). A total of 17 genes were found to be repeated genes, and 79 were found to be unique genes in the chloroplast genomes. Three genes (*clpP, ycf3 and rps12*) contained two introns, whereas 10 genes (*atpF*, *rpoC1*, *rpl2*, *rpl16*, *petB*, *petD*, *trnA-UGC*, *trnI-GAU*, *trnK-UUU* and *trnL-UAA*) had only one intron (Table 2 and Table S2).

### 2.2. Codon Usage Analyses and RNA Editing Sites

**Figure 3:**
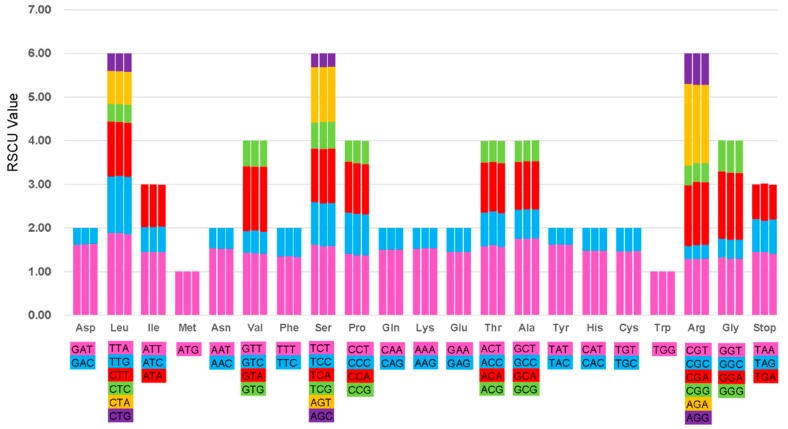
Codon content of 20 amino acids and stop codons in all of the protein-coding genes of the chloroplast genomes of three *Macrosolen* species.

Relative synonymous codon usage (RSCU) is the ratio between the use and expected frequencies for a particular codon and a measure of nonuniform synonymous codon usage in coding sequences [32]. On the basis of the sequences of protein-coding genes, the codon usage frequency was estimated for the chloroplast genome of the three *Macrosolen* species (Figure 3). All the protein-coding genes were found to consist of 21,581, 21,598 and 21,520 codons in the chloroplast genomes of *M. cochinchinensis*, *M. tricolor* and *M. bibracteolatus*, respectively (Table S3). Figure 3 shows that the RSCU value increased with the increase in the quantity of codons which coded for a specific amino acid. Most of the amino acid codons show preferences except for methionine and tryptophan. Potential RNA editing sites were also predicted for 29 genes in the chloroplast genomes of the three species. A total of 39 RNA editing sites were identified (Table S4). The amino acid conversion from serine (S) to leucine (L) occurred most frequently, whereas that from proline (P) to serine (S) and from threonine (T) to methionine (M) occurred the least.

### 2.3. IR Constriction and Expansion

**Figure 4:**
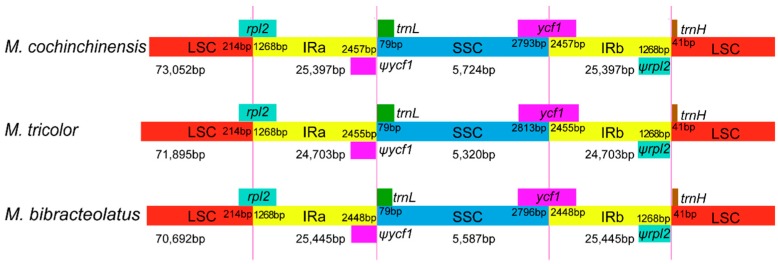
Comparison of the borders of the large single-copy (LSC), small single-copy (SSC), and inverted repeats (IR) regions among the chloroplast genomes of three *Macrosolen* species. The number above the gene features means the distance between the ends of genes and the borders sites. These features are not to scale. Ψ: pseudogenes.

Figure 4 shows the comparison of the boundaries of the LSC/IR/SSC regions of three *Macrosolen* species. The LSC/IR/SSC boundaries and gene contents in the chloroplast genomes of the three species were found to be highly conserved, featuring the same sequence structure and differences in length. In the three species, the *rpl2* gene, which is a normal functional gene, crossed the LSC/IRa boundary, but the *rpl2* pseudogene with a length of 1268 bp formed in the IRb region. The SSC/IRb boundaries of *M. cochinchinensis*, *M. tricolor* and *M. bibracteolatus* were found to be located in the complete *ycf1* gene, and their *ycf1* pseudogenes with lengths of 2457, 2455 and 2448 bp, respectively, were found to be produced in IRa.

### 2.4. Simple Sequence Repeats (SSRs) and Repeat Structure Analyses

**Figure 5:**
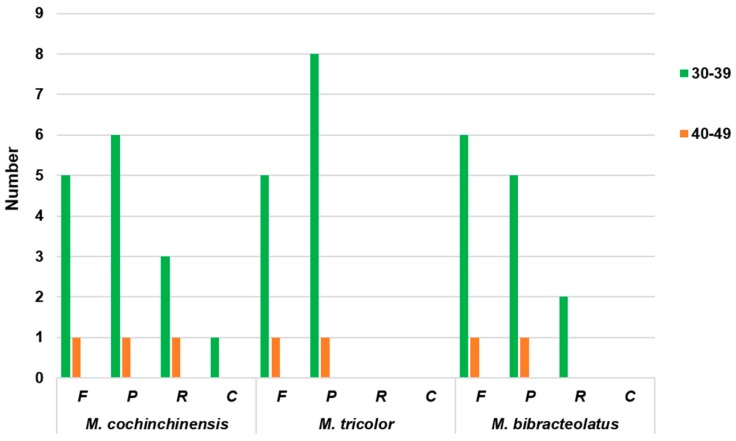
Repeat sequences in the chloroplast genomes of three *Macrosolen* species. F, P, R, and C indicate the repeat types F (forward), P (palindrome), R (reverse) and C (complement), respectively. Repeats with different lengths are indicated in different colors.

A simple sequence repeat (SSR), which is also known as microsatellite DNA, is a tandem repeat sequence consisting of one to six nucleotide repeat units [22]. SSRs are widely used as molecular markers in species identification, population genetics, and phylogenetic investigations due to their high polymorphism level [33,34]. A total of 238, 226 and 217 SSRs were identified in the chloroplast genomes of *M. cochinchinensis*, *M. tricolor* and *M. bibracteolatus*, respectively (Table 3). Amongst all SSRs, the numbers of mononucleotide repeats were the highest, with values detected at 169, 166 and 162 times in *M. cochinchinensis*, *M. tricolor* and *M. bibracteolatus*, respectively. Amongst these mononucleotide repeats, A/T was found to be the most frequent SSR. In accordance with the number of repeats, mononucleotide and dinucleotide SSRs exhibited a certain base preference that mainly contained A/T units. Long repeat sequences should be >30 bp, and these repeats are mainly distributed in the gene spacer and intron sequences. The result shows that *M. cochinchinensis* presented the highest number, comprising six forward, seven palindromic, four reverse and one complement repeats (Figure 5). Two types of *M. tricolor*, comprising six forward and nine palindromic repeats, were present. *M. bibracteolatus* presented seven forward, six palindromic and two reverse repeats.

### 2.5. Comparative Genomic Analyses

**Figure 6:**
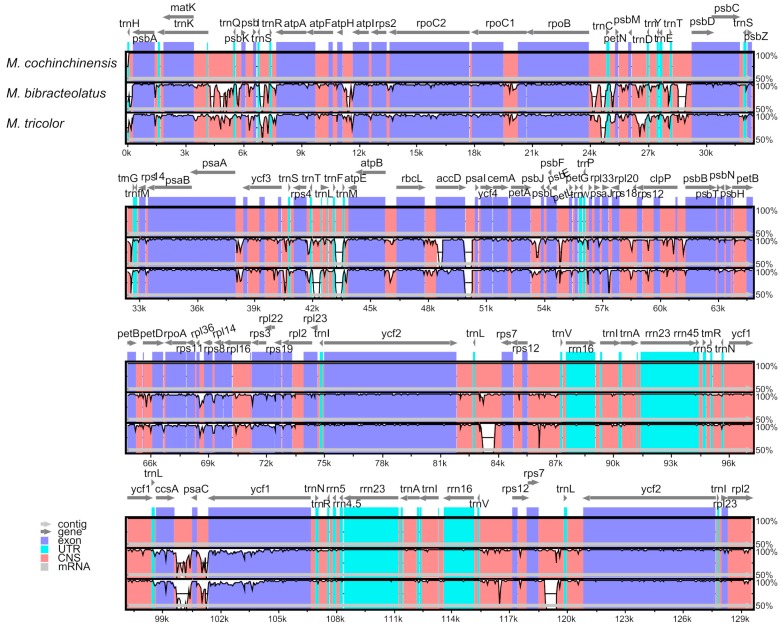
Sequence identity plot comparing the three chloroplast genomes with *M. cochinchinensis* as a reference by using mVISTA. Grey arrows and thick black lines above the alignment indicate genes with their orientation and the position of their IRs, respectively. A cut-off of 70% identity was used for the plots, and the Y-scale represents the percent identity ranging from 50% to 100%.

**Figure 7:**
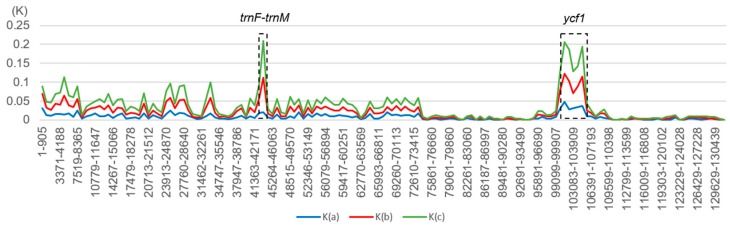
Sliding window analyses of the three whole chloroplast genomes. X-axis: position of a window. Y-axis: sequence divergence between species of each window. K(a): K values between *M. bibracteolatus* and *M. tricolor*; K(b): K values between *M. bibracteolatus* and *M. cochinchinensis*; K(c): K values between *M. tricolor* and *M. cochinchinensis*.

The complete chloroplast of the three chloroplast genomes were compared with that of *M. cochinchinensis* as a reference using the mVISTA program. As shown in Figure 6, the *ycf1* and *ccsA* genes were found to be the most mutant genes. Except for these genes, the other genes were found to be highly conserved, and most of them showed similarities of >90%. The variations in the coding regions were smaller than those in the noncoding regions. Amongst the three chloroplast genomes, the most divergent regions were found to be localized in the intergenic spacers such as *trnF-trnM*. The rRNA genes of the three species were highly conservative, and almost no variations were observed. The K values (sequence divergence between species) were calculated, and the sliding windows of the K values were constructed by the DnaSP [35] (Figure 7). Figure 7 shows that the sequence divergence between *M. tricolor* and *M. cochinchinensis* was much higher than the other two K values. *M. bibracteolatus* and *M. tricolor* showed a small divergence (K < 0.05). The LSC and SSC regions were more divergent than IRs. Two mutational hotspots were found with high K values, and they were located at the LSC and SSC regions. Combined with genes location and the mVISTA result, the two hotspots were found to be *trnF-trnM* and *ycf1*.

### 2.6. Phylogenetic Analyses

**Figure 8:**
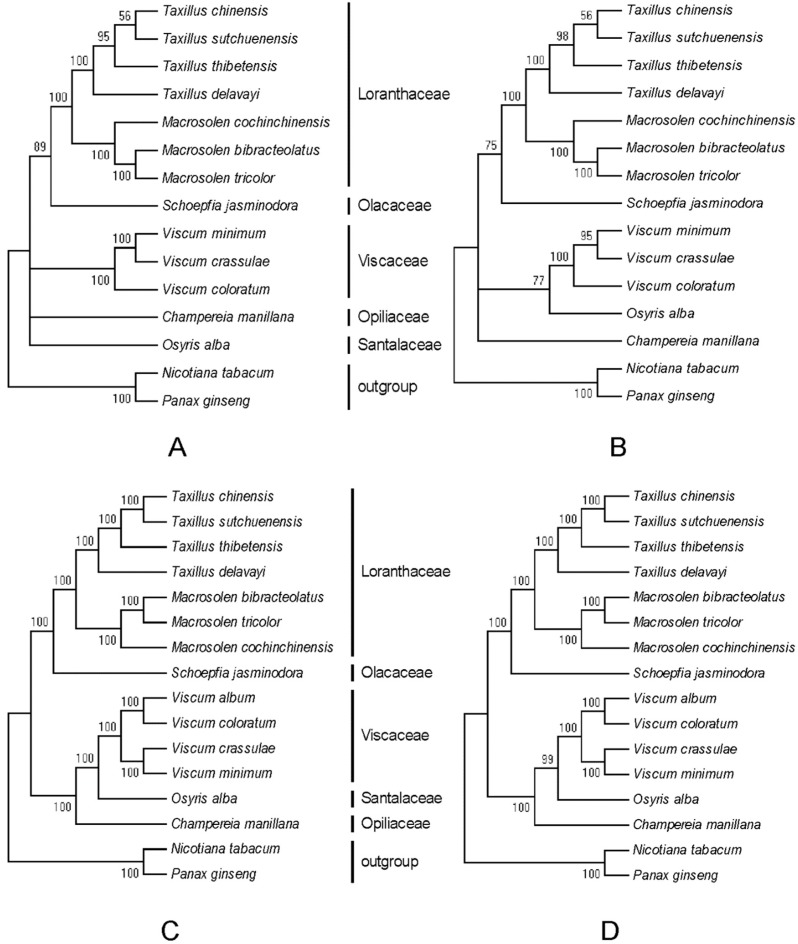
Phylogenetic trees constructed with the *matK* genes of 15 species by using the maximum parsimony (MP) (**A**) and maximum likelihood (ML) (**B**) methods. Phylogenetic trees constructed with 58 common protein-coding genes of 16 species using the MP (**C**) and ML (**D**) methods. Numbers at nodes are bootstrap values.

To analyze the phylogenetic relationships of *Macrosolen* in Santalales, we constructed phylogenetic trees using 58 common protein-coding genes of 16 species and *matK* genes of 15 species by the MP and ML methods with a bootstrap of 1000 repetitions. The MP and ML trees were the same whether they were constructed by either common protein-coding genes or *matK* genes (Figure 8). All nodes in all the phylogenetic trees received a >50% bootstrap value. All four phylogenetic trees showed that the three *Macrosolen* species are sister taxa with respect to *S. jasminodora* (Olacaceae). *M. cochinchinensis*, *M. tricolor* and *M. bibracteolatus* were gathered into one branch with a well-supported bootstrap value (100%). The three species within the genus *Viscum* grouped with *Osyris alba* (Santalaceae) and all Santalales species were clustered within a lineage distinct from the outgroup. As shown in Figure 8, the trees constructed by common protein-coding genes also received a higher bootstrap value than the trees constructed by the *matK* genes.

## 3. Discussion

**Figure 9:**
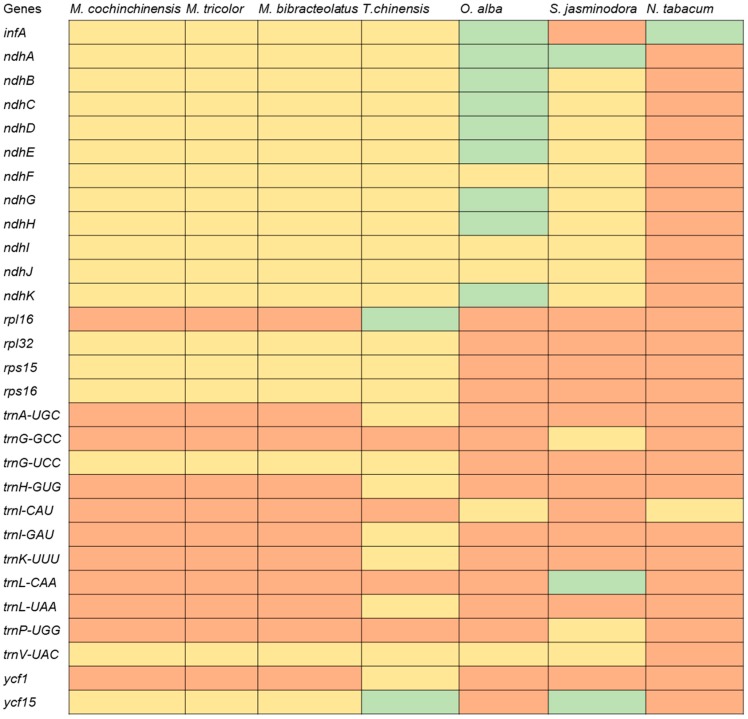
Comparison of the chloroplast genome gene content of six parasitic plants and one model plant (*Nicotiana tabacum*). The common existing genes in the complete chloroplast genome of the seven species are not listed. Red boxes indicate each gene present, and green boxes indicate that each gene is considered as a pseudogene. The yellow boxes indicate an absent gene.

Numerous variations occur in the chloroplast genomes of parasitic plants. However, only a small number of plants within Santalales have been studied. In this study, the complete chloroplast genomes of *M. cochinchinensis*, *M. tricolor* and *M. bibracteolatus* from Santalales were assembled, annotated and analyzed. Compared with the chloroplast genomes of the model plant *Nicotiana tabacum*, all the *ndh* genes of the chloroplast genomes were lost amongst the three species, and the *infA* gene, which codes for a translation initiation factor, was also missing in these species. These cases were similar to those of *T. chinensis* and *T. sutchuenensis* [7]. The *rpl16* and *ycf15* genes were lost in the three species, but they were still present in *T. chinensis* as pseudogenes (Figure 9). However, compared with the results reported by Shin et al. [36], different gene contents of the chloroplast genome were observed in *M. cochinchinensis*. These studies have shown that *M. cochinchinensis* contains the exon 1 fragment of the *ndhB* gene and a fragment of the *infA* gene, whereas the *rpl36* gene is completely lost. However, the *rpl36* gene is still present in the chloroplast genome according to our results. *M. cochinchinensis* has lost the *infA* gene and all *ndh* genes. The number of tRNA genes also differed between the two studies. We annotated 35 tRNA genes, but previous studies only obtained 30 tRNA genes. The evolution of the chloroplast genome in parasitic plants, particularly nonphotosynthetic holoparasites, can lead to significantly reconfigured plastomes [21]. The losses of *ndh* genes are associated with nutritional status or extensive rearrangements of chloroplast structures [37], and they have occurred in the reported chloroplast genomes of parasitic plants [7]. Our study also showed that *ndh* genes were lost in the transformation from autotrophy to heterotrophy [38].

The Santalales order consists of a small number of autotrophic species and a large number of parasitic species which are root or aerial (stem) parasites [39]. According to the Engler system, Santalales consists of seven families. We downloaded five families belonging to Santalales, which were available in the National Center for Biotechnology Information (NCBI) at that time, and two species as outgroups to analyze the phylogenetic relationships of *Macrosolen* in Santalales. The present study showed that Loranthaceae is closely related to Olacaceae, whereas Viscaceae is closely related to Santalaceae and Opiliaceae. These results are similar to those of previous studies [13,14]. All the phylogenetic results strongly support that Loranthaceae and Viscaceae diverged independently from each other.

As folk medicine in China, *M. cochinchinensis*, *M. tricolor* and *M. bibracteolatus* have been used to treat diseases for a long time, and their dried stems and branches with leaves are used as medicinal parts. However, *Macrosolen* species are similar in appearance, especially when they are processed into medicinal slices, thereby causing difficulty in their identification. The identification of parasitic medicinal materials has rarely been reported. Though phytochemical approaches have played an important role in species identification [26], they are inadequate because they are limited to the environment and harvest period. Molecular characterization has shown an improved specificity for plants [23,26]. In our study, mutational hotspots such as the *ycf1* gene, the *ccsA* gene and the *trnF-trnM* intergenic region are potential sites for identification of *Macrosolen* species.

## 4. Materials and Methods

### 4.1. Plant Materials

All the samples in this study were collected from the Guangxi Province of China. Fresh leaves of *M. cochinchinensis* and *M. tricolor* were collected from Qinzhou city, and fresh leaves of *M. bibracteolatus* were collected from Chongzuo city. The three samples were identified by Yonghua Li, who is from the College of Pharmacy, Guangxi University of Traditional Chinese Medicine. The collected fresh leaves were stored in a −80 °C refrigerator until use. 

### 4.2. DNA Extraction, Sequencing and Assembly

All the methods in this article were based on the methods of Zhou et al. [40]. Total genomic DNA was extracted from samples using the DNeasy Plant Mini Kit with a standard protocol (Qiagen Co., Hilden, Germany). The DNA was sequenced according to the manufacturer’s manual for the Illumina Hiseq X. Approximately 6.2 Gb of raw data from *M. cochinchinensis*, 6.5 Gb of raw data from *M. tricolor*, and 6.3 Gb of raw data from *M. bibracteolatus* were generated with 150 bp paired-end read lengths. The software Trimmomatic (version 0.39, Institute for Biology, Aachen, German) [41] was used to filter the low-quality reads of the raw data, and the Q value was defined as Sanger. Then, all the clean reads were mapped to the database on the basis of their coverage and similarity. Burrows–Wheeler Aligner (BWA-MEM, Wellcome Trust Sanger Institute, Wellcome Genome Campus, Cambridge, UK) was used in chloroplast genome assembly to generate the bam files. The depth was calculated using Samtools (Medical Population Genetics Program, Broad Institute, Cambridge, MA, USA) and plotted using Rscript (with the smoothScatter function). The accuracy of the assembly of the four boundaries (SSC, LSC and IR regions) of the chloroplast sequences was confirmed through PCR and Sanger sequencing using the validated primers listed in Table S5. The assembled complete chloroplast genome sequence of *M. cochinchinensis*, *M. tricolor* and *M. bibracteolatus* were submitted to the NCBI, and the accession numbers were MH161424, MH161425 and MH161423, respectively. The raw data of three species were submitted to the NCBI. The Bioproject ID of this study is PRJNA587349. The SRA accession ID of *M. tricolor* is SRR10442639, that of *M. bibracteolatus* is SRR10442640, and that of *M. cochinchinensis* is SRR10442641.

### 4.3. Genome Comparison and Phylogenetic Analyses

The whole-genome alignment for the chloroplast genomes of three *Macrosolen* species were performed and plotted using the mVISTA program (http://genome.lbl.gov/vista/mvista/submit.shtml) [42]. Gene content comparison was analyzed by CPGAVAS2 (Institute of Medicinal Plant Development, Chinese Academy of Medical Sciences and Peking Union Medical College, Beijing, China) [43] and identified by manual correction. To determine the phylogenetic positions of three *Macrosolen* species within Santalales, we analyzed the chloroplast genomes of 16 species, encompassing 11 other taxa within this lineage, *Viscum album* (KT003925), *V. coloratu* (NC_035414), *V. crassula* (KT070881), *V. minimum* (KJ512176), *Osyris alba* (KT070882), *Schoepfia jasminodora* (KX775962), *Champereia manillana* (NC_034931), *T. chinensis* (KY996492), *T. sutchuenensis* (KY996493), *T. delavayi* (MH161426), and *T. thibetensis* (MH161427). The chloroplast genomes of *Panax ginseng* (AY582139) and *N. tabacum* (NC_001879) were used as outgroups.

### 4.4. Other Analyses

On the basis of the study of Zhou et al. [40], we analyzed the complete chloroplast genome of three *Macrosolen* species, including genome structure analyses (genome length, gene content and GC content), codon usage analyses, RNA editing site prediction, and repeat sequences analyses. The distribution of codon usage was investigated using the CodonW software (University of Texas, Houston, TX, USA) with the RSCU ratio [32]. Potential RNA editing sites were predicted using the Predictive RNA Editor for Plants (PREP-Cp, Center for Plant Science Innovation, University of Nebraska-Lincoln, Lincoln, NE, USA) suite online program [44] with a cutoff value of 0.8. Simple sequence repeats were detected using the MISA software (Pgrc.ipk-gatersleben.de/misa/) [45]. Repeat sequences were identified by REPuter (University of Bielefeld, Bielefeld, Germany) [46].

## Figures and Tables

**Table 1:** Length of chloroplast genome of three *Macrosolen* species and their base composition.

Species	*M. cochinchinensis*	*M. tricolor*	*M. bibracteolatus*
Accession No.	MH161424	MH161425	MH161423
Genome size (bp)	129,570	126,621	127,169
LSC length (bp)	73,052	71,895	70,692
SSC length (bp)	5724	5320	5587
IRs length (bp)	25,397	24,703	25,445
GC content (%)	37.3	37.7	37.9
Number of genes	113	113	113
Number of protein-coding genes	68	68	68
Number of tRNAs	35	35	35
Number of rRNAs	8	8	8
Number of pseudogenes	2	2	2

**Table 2:** Gene list of chloroplast genome of three *Macrosolen* species.

No.	Group of Genes	Gene Names	Number
1	Photosystem I	*psaA*, *psaB*, *psaC*, *psaI*, *psaJ*	5
2	Photosystem II	*psbA*, *psbB*, *psbC*, *psbD*, *psbE*, *psbF*, *psbH*, *psbI*, *psbJ*, *psbK*, *psbL*, *psbM*, *psbN*, *psbT*, *psbZ*	15
3	Cytochrome b/f complex	*petA*, petB *, petD *, *petG*, *petL*, *petN*	6
4	ATP synthase	*atpA*, *atpB*, *atpE*, atpF *, *atpH*, *atpI*	6
5	NADH dehydrogenase	-	0
6	RubisCO large subuni	*rbcL*	1
7	RNA polymerase	*rpoA*, *rpoB*, rpoC1 *, *rpoC2*	4
8	Ribosomal proteins (SSU)	*rps2*, *rps3*, *rps4*, *rps7 (×2)*, *rps8*, *rps11*, rps12 ** (×2), *rps14*, *rps18*, *rps19*	12(2)
9	Ribosomal proteins (LSU)	rpl2 * (×2), *rpl14*, rpl16 *, *rpl20*, *rpl22*, *rpl23 (×2)*, *rpl33*, *rpl36*	10(2)
10	Proteins of unknown function	*ycf1(×2)*, *ycf2(×2)*, ycf3 **, *ycf4*	6(2)
11	Transfer RNAs	*35 tRNAs* (4 contain an intron, 7 in the IRs)	35(7)
12	Ribosomal RNAs	*rrn4.5 (×2)*, *rrn5(×2)*, *rrn16 (×2)*, *rrn23 (×2)*	8(4)
13	Other genes	*accD*, clpP **, *matK*, *ccsA*, *cemA*	5

* One or two asterisks following genes indicate one or two contained introns, respectively. (×2) indicates that the number of the repeat unit is two. The numbers in parenthesis at the line of ‘Number’ indicate the total number of repeated genes.

**Table 3:** Types and amounts of simple sequence repeats (SSRs) in the chloroplast genomes of three *Macrosolen* species.

SSR Type	Repeat Unit	Amount			Ratio (%)		
		①	②	③	①	②	③
mono	A/T	161	159	153	95.3	95.8	94.4
	C/G	8	7	9	4.7	4.2	5.6
di	AC/GT	5	4	4	9.6	8.5	9.3
	AG/CT	13	14	13	25	29.8	30.2
	AT/TA	34	29	26	64.4	61.7	60.5
tri	AAT/ATT	4	4	0	66.7	66.7	0
	ATC/ATG	2	2	2	33.3	33.3	100
tetra	AAAG/CTTT	3	3	3	33.3	42.9	30
	AATC/ATTG	1	1	0	11.1	14.3	0
	ACAG/CTGT	1	1	1	11.1	14.3	10
	AAAT/ATTT	3	1	3	33.3	14.3	30
	AATG/ATTC	1	0	1	11.1	0	10
	AGAT/ATCT	0	1	1	0	14.3	10
	ACAT/ATGT	0	0	1	0	0	10
penta	AATAT/ATATT	1	0	0	100	0	0
hexa	ATATCC/ATATGG	1	0	0	100	0	0

① *M. cochinchinensis*; ② *M. tricolor*; and ③ *M. bibracteolatus*.

## Supplementary Materials

Supplementary materials can be found at https://www.mdpi.com/1422-0067/20/22/5812/s1.

## Abbreviations

LSC	Large single copy
SSC	Small single copy
IR	Inverted repeat
MP	Maximum parsimony
ML	Maximum likelihood
RSCU	Relative synonymous codon usage
SSR	Simple Sequence Repeats
NCBI	National Center for Biotechnology Information
