# Supplementary material for: Gene Losses and Variations in Chloroplast Genome of Parasitic Plant Macrosolen and Phylogenetic Relationships within Santalales
Source: Int J Mol Sci. 2019 Nov 19;20(22):5812. doi: 10.3390/ijms20225812 (PMC6888684; doi:10.3390/ijms20225812)
Supplement: Supplementary file 1 [file ijms-20-05812-s001.pdf]

Supplementary Tables

Table S1. Gene content and gene order of the chloroplast genomes of three *Macrosolen* species

Species	Regions	Positions	T(U) (%)	C (%)	A (%)	G (%)	Length (bp)
Macrosolen	Total		31.9	19.0	30.8	18.3	129,570
cochinchinensis	IRa		29.0	22.0	28.8	20.3	25,397
	IRb		28.8	20.3	29.0	22.0	25,397
	LSC		33.4	18.0	31.8	16.9	73,052
	SSC		40.6	12.5	35.3	11.6	5,724
	CDS		31.3	17.3	31.4	19.9	64,743
		1st position	23.8	18.5	31.4	26.2	21,581
		2nd position	32.3	19.6	30.7	17.4	21,581
		3rd position	37.9	13.9	32.1	16.1	21,581
Macrosolen	Total		31.8	19.2	30.5	18.5	126,621
tricolor	IRa		29.0	22.1	28.6	20.3	24,703
	IRb		28.6	20.3	29.0	22.1	24,703
	LSC		33.3	18.2	31.5	17.0	71,895
	SSC		40.3	13.4	33.8	12.4	5,320
	CDS		31.3	17.4	31.3	20.0	64,794
		1st position	23.8	18.6	31.3	26.2	21,598
		2nd position	32.3	19.6	30.6	17.5	21,598
		3rd position	37.7	13.9	32.1	16.2	21,598
Macrosolen	Total		31.7	19.3	30.5	18.6	127,169
bibracteolatus	IRa		29.0	22.1	28.7	20.3	25,445
	IRb		28.7	20.3	29.0	22.1	25,445
	LSC		33.1	18.4	31.4	17.1	70,692
	SSC		39.8	13.4	34.0	12.7	5,587
	CDS		31.2	17.4	31.3	20.0	64,560
		1st position	23.8	18.8	31.3	26.2	21,520
		2nd position	32.3	19.6	30.6	17.5	21,520
		3rd position	37.6	13.9	32.1	16.4	21,520

1. CDS: protein-coding regions; 2. 1st position: 1st base of codons; 3. 2nd position: 2nd base of codons; 4. 3rd position: 3rd base of codons.

Table S2. Genes content introns of three *Macrosolen* species

Species	Gene	Location	Exon1(bp)	Intron1(bp)	Exon2(bp)	Intron2(bp)	Exon3(bp)
M. cochinchinensis	*atpF*	LSC	150	750	405		
	clpP	LSC	71	838	292	617	228
	petB	LSC	6	755	642		
	petD	LSC	5	538	478		

	rpl2	LSC, IR	395	655	433		
	rpl16	LSC	9	990	399		
	rpoC1	LSC	432	749	1,614		
	rps12	LSC, IR	114	--	232	520	26
	trnA-UGC	IR	38	815	35		
	trnI-GAU	IR	35	912	37		
	trnK-UUU	LSC	37	2,473	35		
	trnL-UAA	LSC	37	291	50		
	ycf3	LSC	125	726	229	698	153
M. tricolor	*atpF*	LSC	150	745	405		
	clpP	LSC	71	828	292	594	228
	petB	LSC	6	758	642		
	petD	LSC	5	556	478		
	rpl2	LSC, IR	395	656	433		
	rpl16	LSC	9	1,003	399		
	rpoC1	LSC	432	721	1,614		
	rps12	LSC, IR	114	--	232	531	26
	trnA-UGC	IR	38	815	35		
	trnI-GAU	IR	35	909	37		
	trnK-UUU	LSC	37	2,497	35		
	trnL-UAA	LSC	37	283	50		
	ycf3	LSC	125	728	229	680	153
M. bibracteolatus	*atpF*	LSC	150	744	405		
	clpP	LSC	71	790	292	598	228
	petB	LSC	6	752	642		
	petD	LSC	5	532	478		
	rpl2	LSC, IR	395	656	433		
	rpl16	LSC	9	990	399		
	rpoC1	LSC	432	721	1,614		
	rps12	LSC, IR	114	--	232	531	26
	trnA-UGC	IR	38	814	35		
	trnI-GAU	IR	35	909	37		
	trnK-UUU	LSC	37	2,490	35		
	trnL-UAA	LSC	37	278	50		
	ycf3	LSC	125	758	229	689	153

Table S3. Codon usage of three *Macrosolen* species

Amino acid	Codon	No.			RSCU			Amino acid	Codon	No.			RSCU		
		①	②	③	①	②	③			①	②	③	①	②	③
Phe	TTT	874	876	860	1.35	1.36	1.34	Tyr	TAT	631	629	626	1.62	1.63	1.62
Phe	TTC	421	414	422	0.65	0.64	0.66	Tyr	TAC	149	143	145	0.38	0.37	0.38
Leu	TTA	697	700	686	1.89	1.89	1.86	Stop	TAA	33	33	32	1.46	1.46	1.41
Leu	TTG	481	486	489	1.30	1.31	1.32	Stop	TAG	17	16	18	0.75	0.71	0.79
Leu	CTT	466	458	459	1.26	1.24	1.24	Stop	TGA	18	19	18	0.79	0.84	0.79

Leu	CTC	146	150	151	0.40	0.40	0.41	Trp	TGG	368	372	370	1.00	1.00	1.00
Leu	CTA	279	282	282	0.76	0.76	0.76	Gln	CAA	603	605	611	1.51	1.52	1.52
Leu	CTG	143	147	150	0.39	0.40	0.41	Gln	CAG	198	189	192	0.49	0.48	0.48
Ile	ATT	894	889	887	1.46	1.46	1.46	Asn	AAT	827	823	807	1.54	1.53	1.53
Ile	ATC	350	348	354	0.57	0.57	0.58	Asn	AAC	246	250	249	0.46	0.47	0.47
Ile	ATA	592	593	577	0.97	0.97	0.95	Lys	AAA	1,005	1,010	1,006	1.53	1.54	1.54
Met	ATG	477	475	479	1.00	1.00	1.00	Lys	AAG	311	304	303	0.47	0.46	0.46
Val	GTT	411	411	407	1.44	1.43	1.42	Asp	GAT	737	745	732	1.62	1.63	1.64
Val	GTC	141	148	144	0.49	0.52	0.50	Asp	GAC	173	170	163	0.38	0.37	0.36
Val	GTA	427	420	427	1.49	1.46	1.49	Glu	GAA	824	820	823	1.46	1.46	1.46
Val	GTG	166	170	168	0.58	0.59	0.59	Glu	GAG	302	305	306	0.54	0.54	0.54
Ser	TCT	441	433	433	1.62	1.58	1.59	Pro	CCT	301	295	295	1.42	1.38	1.38
Ser	TCC	267	270	268	0.98	0.99	0.99	Pro	CCC	199	204	200	0.94	0.95	0.94
Ser	TCA	334	342	339	1.23	1.25	1.25	Pro	CCA	247	248	246	1.17	1.16	1.15
Ser	TCG	164	171	169	0.60	0.62	0.62	Pro	CCG	100	109	111	0.47	0.51	0.52
Ser	AGT	344	342	341	1.26	1.25	1.25	Arg	CGT	283	285	288	1.29	1.29	1.30
Ser	AGC	82	84	82	0.30	0.31	0.30	Arg	CGC	66	70	70	0.30	0.32	0.32
Cys	TGT	177	176	176	1.47	1.47	1.48	Arg	CGA	308	321	316	1.40	1.45	1.43
Cys	TGC	64	63	62	0.53	0.53	0.52	Arg	CGG	98	97	100	0.45	0.44	0.45
Thr	ACT	432	437	428	1.58	1.61	1.57	Arg	AGA	411	395	397	1.87	1.79	1.79
Thr	ACC	213	213	212	0.78	0.78	0.78	Arg	AGG	151	157	158	0.69	0.71	0.71
Thr	ACA	315	307	311	1.15	1.13	1.14	His	CAT	421	424	421	1.49	1.49	1.49
Thr	ACG	131	132	137	0.48	0.48	0.50	His	CAC	144	144	145	0.51	0.51	0.51
Ala	GCT	479	476	476	1.76	1.76	1.77	Gly	GGT	462	454	453	1.33	1.3	1.30
Ala	GCC	179	181	178	0.66	0.67	0.66	Gly	GGC	150	155	154	0.43	0.44	0.44
Ala	GCA	300	299	298	1.10	1.11	1.11	Gly	GGA	535	539	534	1.54	1.54	1.53
Ala	GCG	129	124	126	0.47	0.46	0.47	Gly	GGG	247	251	253	0.71	0.72	0.73

① *M. cochinchinensis*; ② *M. tricolor*; ③ *M. bibracteolatus*

Table S4. Potential RNA editing sites of three *Macrosolen* species

Gene	Nucleotide Position			Codon change	Amino acid change	Score
	①	②	③			
*acc*D	-	-	-	-	-	-
*atp*A	773	773	773	TCA-TTA	S-L	1
	914	914	914	TCA-TTA	S-L	1
	1,148	1,148	1,148	TCA-TTA	S-L	1
*atp*B	-	-	-	-	-	-
*atp*F	92	92	92	CCA-CTA	P-L	0.86
*atp*I	23	23	23	ACC-ATC	T-I	1
	629	629	629	TCA-TTA	S-L	1
*ccs*A	137	137	137	ACA-ATA	T-I	1
*clp*P	559	559	559	CAT-TAT	H-Y	1
*mat*K	322	322	322	CTT-TTT	L-F	0.86

	646	646	646	CAT-TAT	H-Y	1
	1,384	1,384	1,384	CCG-TCG	P-S	0.86
	-	1433	-	TCG-TTG	S-L	1
*pet*B	418	418	418	CGG-TGG	R-W	1
	611	611	611	TCA-TTA	S-L	1
*pet*D	-	-	-	-	-	-
*pet*G	-	-	-	-	-	-
*pet*L	-	-	-	-	-	-
*psa*B	-	-	-	-	-	-
*psa*I	-	-	-	-	-	-
*psb*B	305	305	305	GCA-GTA	A-V	0.86
*psb*E	-	-	-	-	-	-
*psb*F	-	-	-	-	-	-
*psb*L	2	-	-	ACG-ATG	T-M	1
*rpl*2	-	-	-	-	-	-
*rpl*20	308	308	308	TCA-TTA	S-L	0.86
*rpl*23	-	-	-	CAT-TAT	H-Y	1
*rpo*A	368	368	368	TCG-TTG	S-L	1
	745	745	745	CTT-TTT	L-F	0.86
*rpo*B	338	338	338	TCT-TTT	S-F	1
	430	430	430	CCG-TCG	S-L	0.86
	-	473	473	TCA-TTA	S-L	0.86
	551	551	551	TCA-TTA	S-L	1
	566	566	566	TCG-TTG	S-L	1
	898	898	898	CTT-TTT	L-F	0.86
	2,342	-	2,342	ACA-ATA	T-I	1
*rpo*C1	-	41	41	TCG-TTG	S-L	1
	-	1,762	1,762	CAT-TAT	H-Y	0.86
*rpo*C2	815	815	815	GCC-GTC	A-V	0.86
	827	-	-	ACA-ATA	T-I	1
	1,256	1,256	1,256	GCG-GTG	A-V	0.86
	3,686	3,686	3,692	TCA-TTA	S-L	0.86
	4,064	4,076	4,082	TCT-TTT	S-F	0.8
*rps*2	248	248	248	TCA-TTA	S-L	1
*rps*8	92	92	92	ACC-ATC	T-I	1
	182	182	182	TCA-TTA	S-L	1.86
*rps*14	80	80	80	TCA-TTA	S-L	1
	149	149	149	CCA-CTA	P-L	1
*ycf*3	-	-	-	-	-	-

① *M. cochinchinensis;* ② *M. tricolor;* ③ *M. bibracteolatus*

Table S5. Primer sequences at the boundaries between SC and IR regions of three *Macrosolen* species

Species	Regions	Forward/Reverse	Primer sequence (5′ to 3′)
M. cochinchinensis	LSC-IRa	F	TCCGCCTTTGTGTTAAGCTT
		R	CGGTCTGAATGCTACATGAAGA
	IRa-SSC	F	GCGCACTCAAAGGCGTAAAA
		R	AAAACCTGCTGTCATGCCCT
	SSC-IRb	F	AACCGTTGTGATGGGAAAGACA
		R	AAAGGTTCCATGCGCACTCA
	IRb-LSC	F	CGGTCTGAATGCTACATGAAGA
		R	CCGTGCTAACCTTGGTATGG
M. bibracteolatus	LSC-IRa	F	GTAGATGCTCGGGACCAAGT
		R	ACATAAGCCAGATGACGGAAC
	IRa-SSC	F	TGATCAAGCAGAGGAGGTGG
		R	GGAGAAACCCCATGAAAGGA
	SSC-IRb	F	ACCGTTGGGATGTGAAAGACA
		R	GCGCACTCAAAGGCGTAAAA
	IRb-LSC	F	ACATAAGCCAGATGACGGAAC
		R	TGGTTCACAGCCTTAGGTATCA
M. tricolor	LSC-IRa	F	CCCCTTCCGTTATAGATCGAAA
		R	CCATGAGTGATTTGGCAGATTT
	IRa-SSC	F	CAAGCAGAGGAGGTGGCTTT
		R	CTGCTGTCATGCCCTTTTCTG
	SSC-IRb	F	AAACCGTTGTGATGGGAAAGACA
		R	TGCGCACTCAAAGGCGTAAA
	IRb-LSC	F	ACATAAGCCAGATGACGGAAC
		R	CCGTGCTAACCTTGGTATGG

The thermocycler conditions for the PCR:

94°C 5min;

94°C 30s, 56°C 30s, 72°C 45s, 40 cycles;

72°C 10min.
